# Supplementary figures and images for: Evolution of the WRKY Family in Angiosperms and Functional Diversity under Environmental Stress
Source: Int J Mol Sci. 2024 Mar 21;25(6):3551. doi: 10.3390/ijms25063551 (PMC10971295; doi:10.3390/ijms25063551)

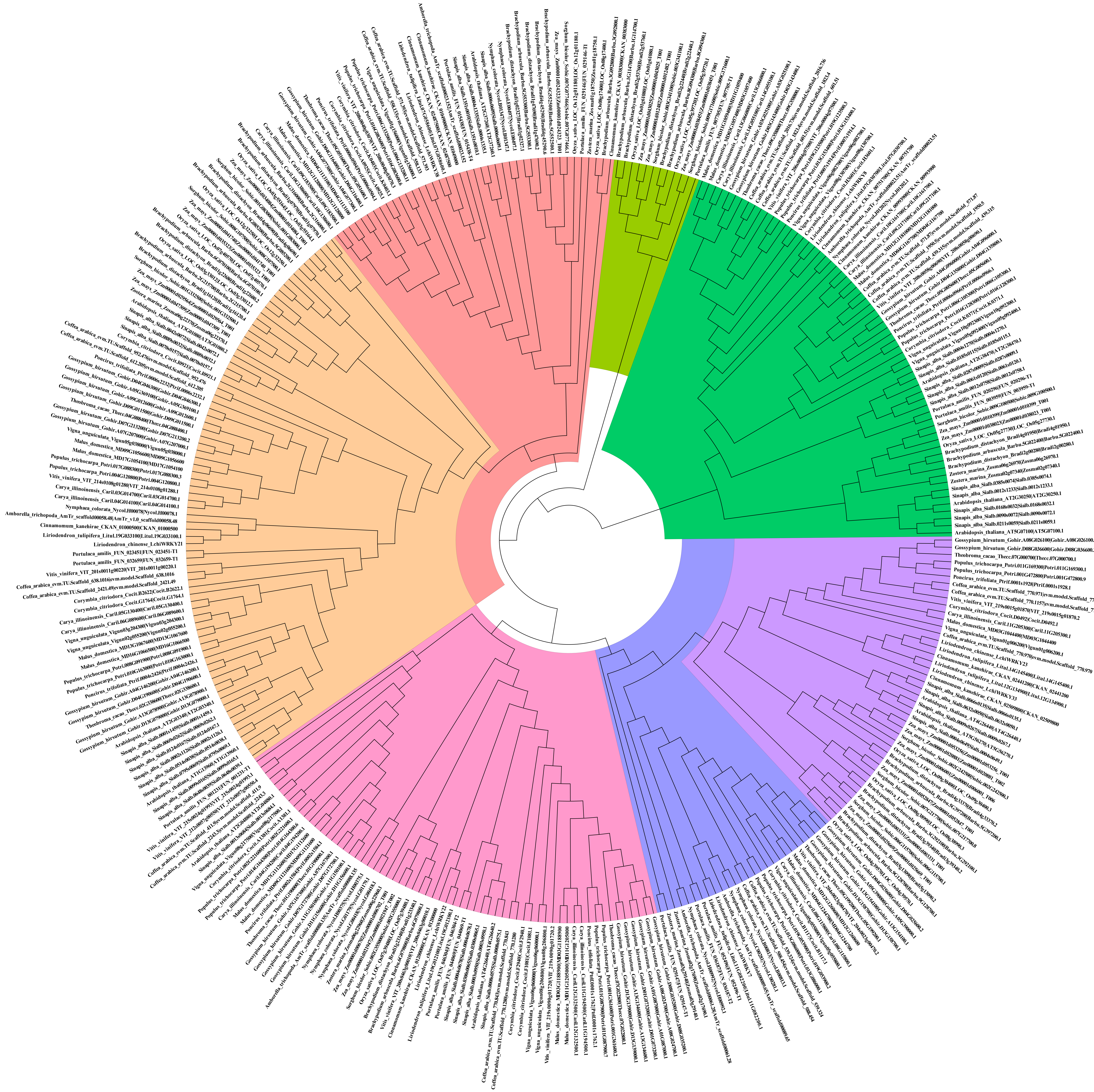

Supplement: Supplementary file 1 [file ijms-25-03551-s001.zip › Figure S1 24-species-WRKY-singlecopy-genetree.pdf]

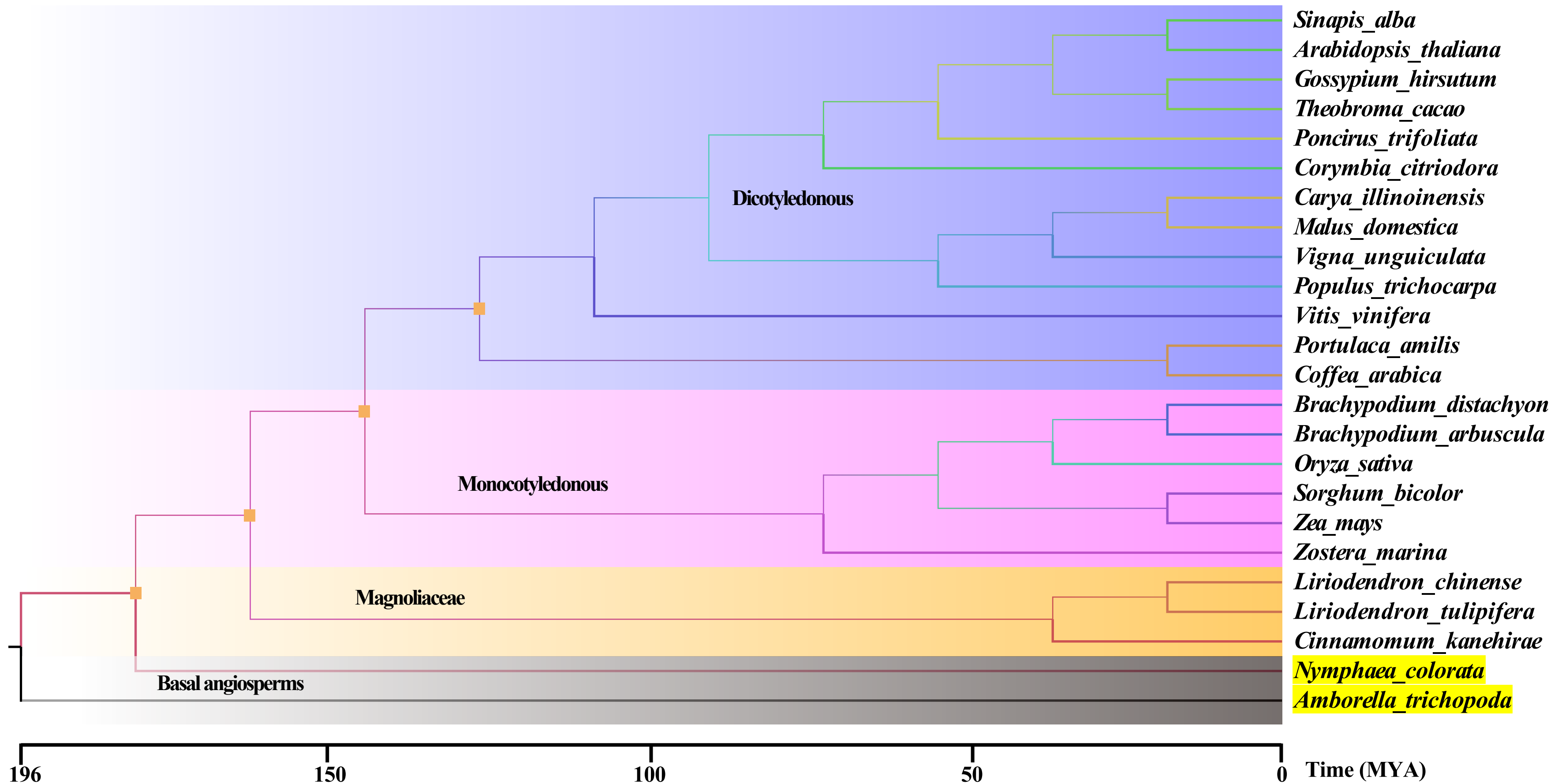

Supplement: Supplementary file 1 [file ijms-25-03551-s001.zip › Figure S2 24-species-list.nwk.pdf]
